# A Case of Granulomatosis with Polyangiitis: Consequences of Delayed Diagnosis in a Life-threatening Malady

**DOI:** 10.7759/cureus.6182

**Published:** 2019-11-18

**Authors:** Amir A Razmjou, Young-Ji Seo, Michael F Ayoub, Jonathan Zuckerman, Satya Patel

**Affiliations:** 1 Internal Medicine, University of California Los Angeles, Los Angeles, USA; 2 Pathology and Laboratory Medicine, University of California Los Angeles, Los Angeles, USA

**Keywords:** anca vasculitis, granulomatosis with polyangiitis, wegener granulomatosis

## Abstract

Granulomatosis with polyangiitis (GPA) is one of three described anti-neutrophil cytoplasmic antibody (ANCA)-associated vasculitides (AAV). Early diagnosis and treatment of GPA is paramount, as it may help prevent irreversible end-organ damage, especially renal and pulmonary failure.

A 72-year-old male with a past medical history of lung adenocarcinoma in remission, chronic sinusitis status-post multiple sinus surgeries, and coronary artery disease presented with shortness of breath, dark urine, and asymmetric polyarthralgias. He had an acute kidney injury, leukocytosis, with urinalysis demonstrating pyuria and hematuria, without casts. Chest imaging showed cavitary nodular opacities in addition to interval increase of existing nodules compared to the most recent scan one month prior. His acute kidney injury progressed to renal failure requiring hemodialysis, and he developed an inflammatory polyarthritis. GPA was suspected clinically so he was started on high-dose intravenous corticosteroids, and subsequently plasmapheresis and rituximab. Serology returned with highly positive proteinase-3 antibodies, and cytoplasmic ANCA positivity on immunofluorescence. Renal biopsy demonstrated severely active pauci-immune glomerulonephritis. Several months after discharge, the patient passed away from gram positive bacteremia.

This patient’s recurrent sinusitis, pulmonary nodules, and subsequent renal failure were highly suggestive of GPA. A biopsy is recommended to confirm the diagnosis of GPA, but treatment should not be delayed if there is a high index of suspicion for the disease. Induction therapy with corticosteroids combined with rituximab or cyclophosphamide has significantly decreased the mortality of patients with GPA.

Patients with GPA often have preceding history of nasopharyngeal and upper airway disease, and can present with fluctuating pulmonary infiltrates. Early recognition and treatment of patients with GPA can prevent life-threatening complications and reduce mortality.

## Introduction

Granulomatosis with polyangiitis (GPA) is one of three described anti-neutrophil cytoplasmic antibody (ANCA)-associated vasculitides (AAV). The pathogenesis of GPA is believed to arise from an environmental or infectious trigger in a genetically predisposed individual, leading to production of ANCAs, pathogenic autoantibodies which can lead to this necrotizing small and medium-vessel vasculitis [1-2]. In vitro evidence suggests that neutrophils are ‘primed’ by cytokines, which leads to translocation of ANCA antigens from cytoplasmic granules, to the cell surface. ANCAs then bind to antigens on the neutrophilic cellular membrane which leads to neutrophil activation and infiltration into the vessel wall leading to end-organ damage [2]. The interaction of ANCAs and neutrophils leads to a complex inter-play with the complement system, monocytes/macrophages (with subsequent necrotizing granuloma formation), and T-cells [1-2]. The hallmark finding on pathology is necrotizing granulomatous inflammation of the upper and lower airways, and necrotizing small and medium-vessel vasculitis [3]. Some clinical manifestations of GPA include nasal, oral, or tracheal ulcers, rhinosinusitis, pulmonary disease/respiratory failure, renal failure, cutaneous vasculitis, and non-specific systemic symptoms. Diagnosis and treatment of GPA in its early stages is critical, as it may help prevent irreversible end-organ damage [4].

## Case presentation

A 72-year-old male with a past medical history of lung adenocarcinoma status-post partial lobectomy four years prior (in remission), chronic sinusitis status-post multiple sinus surgeries four years prior (maxillary antrostomies, ethmoidectomies, and frontal sinusotomies), and coronary artery disease status-post coronary artery bypass graft surgery presented with shortness of breath and dark urine for several days, as well as progressive asymmetric arthralgias in his elbows, hands, and knees. Pertinent medications included fluticasone nasal spray, lisinopril, aspirin, and ibuprofen. The patient had a 15 pack-year smoking history (quit with cancer diagnosis), and did not use illicit drugs or drink alcohol. Family history was non-contributory.

He had presented to the emergency department (ED) 12 days prior for fatigue, sore throat, cough, and shortness of breath, and was diagnosed with community-acquired pneumonia and discharged with a 10-day supply of doxycycline. Tuberculosis interferon-gamma release assay and coccidioides antibody assay were both negative. The patient finished his course of antibiotics, but his shortness of breath continued to worsen. He subsequently developed cola-colored urine, at which point he re-presented to the ED.

**Figure 1:**
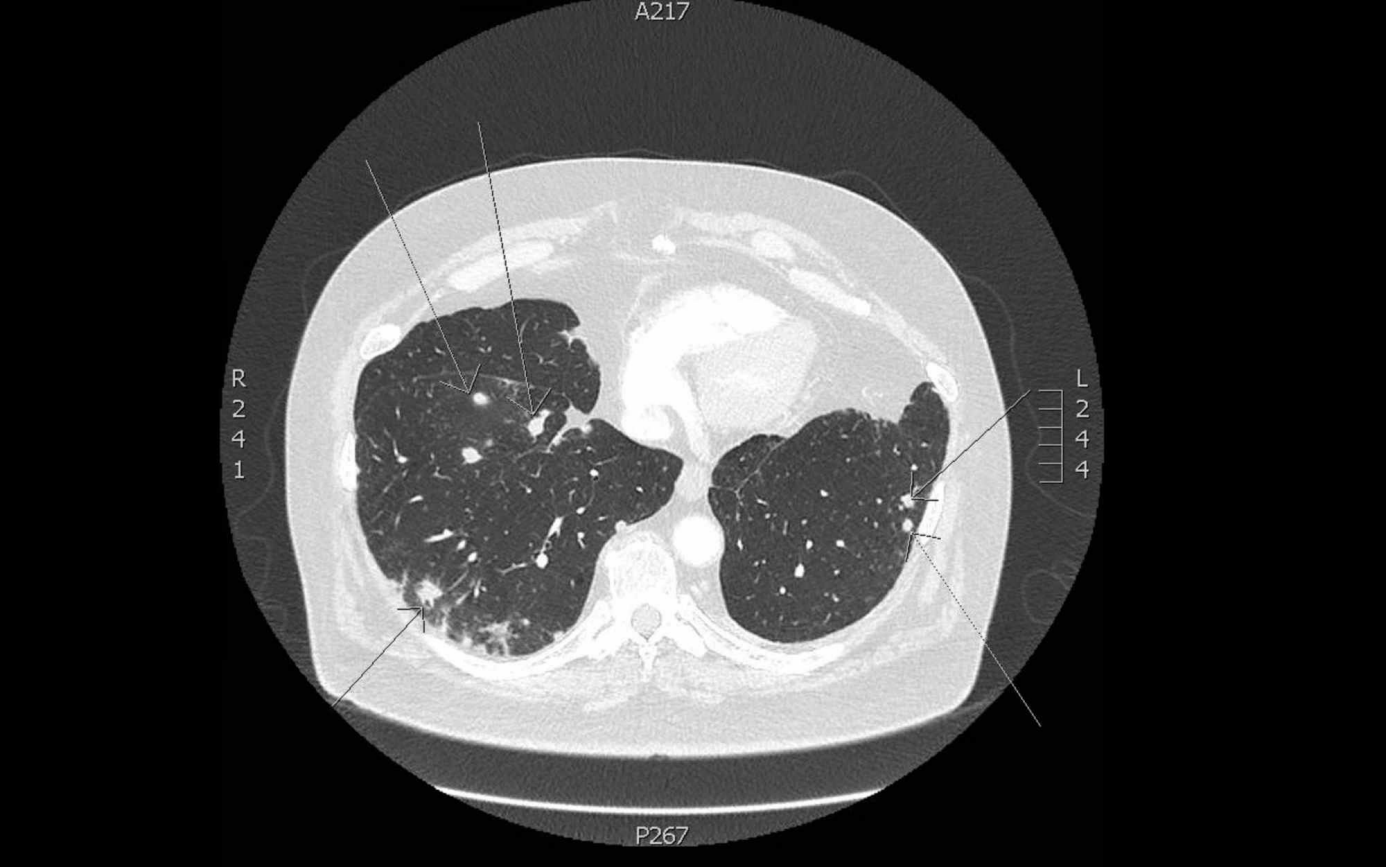
CT chest demonstrating numerous cavitary nodular opacities (arrows). CT: Computed tomography

Vitals in the ED were as follows: temperature of 98.8 degrees Fahrenheit, heart rate of 109 beats per minute, respiratory rate of 20 breaths per minute, blood pressure of 128/75 mmHg, and oxygen saturation of 100% on room air. His serum creatinine (Cr) was 1.74 mg/dL (baseline of 1.0 mg/dL) and white blood cell (WBC) count was 11.82 k/uL with 86% neutrophils. His urinalysis (UA) showed > 182 red blood cells (RBC) per high powered field (HPF), 52 WBCs per HPF, few urine bacteria, urine protein > 500 mg/dL and no casts. Of note, due to his lung cancer surveillance, he had multiple previous computed tomography (CT) scans of his chest. A scan two years prior showed multilobular nodules with cavitation, which when biopsied were negative for recurrence of malignancy and did not demonstrate any infectious etiologies. A repeat scan six months later demonstrated resolution of these prior lesions. The latest scan one month prior to presentation demonstrated recurrent pulmonary nodules. A CT scan in the ED (Figure 1) showed new cavitary nodular opacities in addition to existing nodules with interval increase in size compared to the most recent scan one month prior.

**Figure 2:**
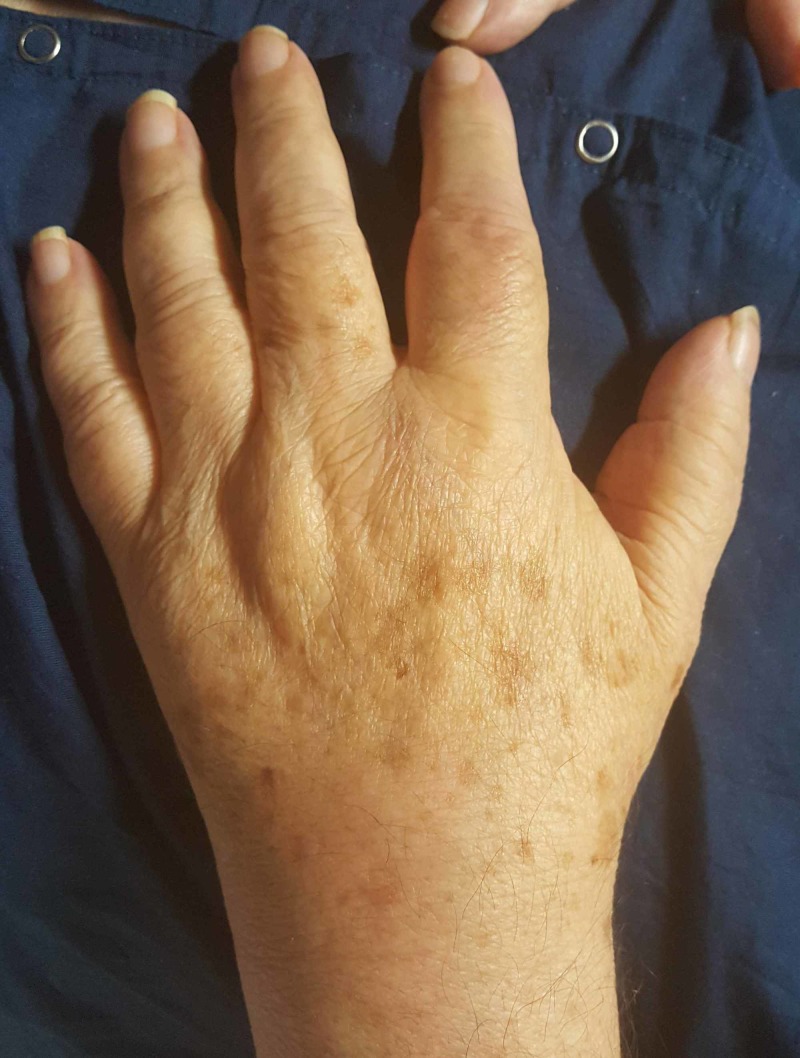
Left wrist with swelling and effusion.

**Figure 3:**
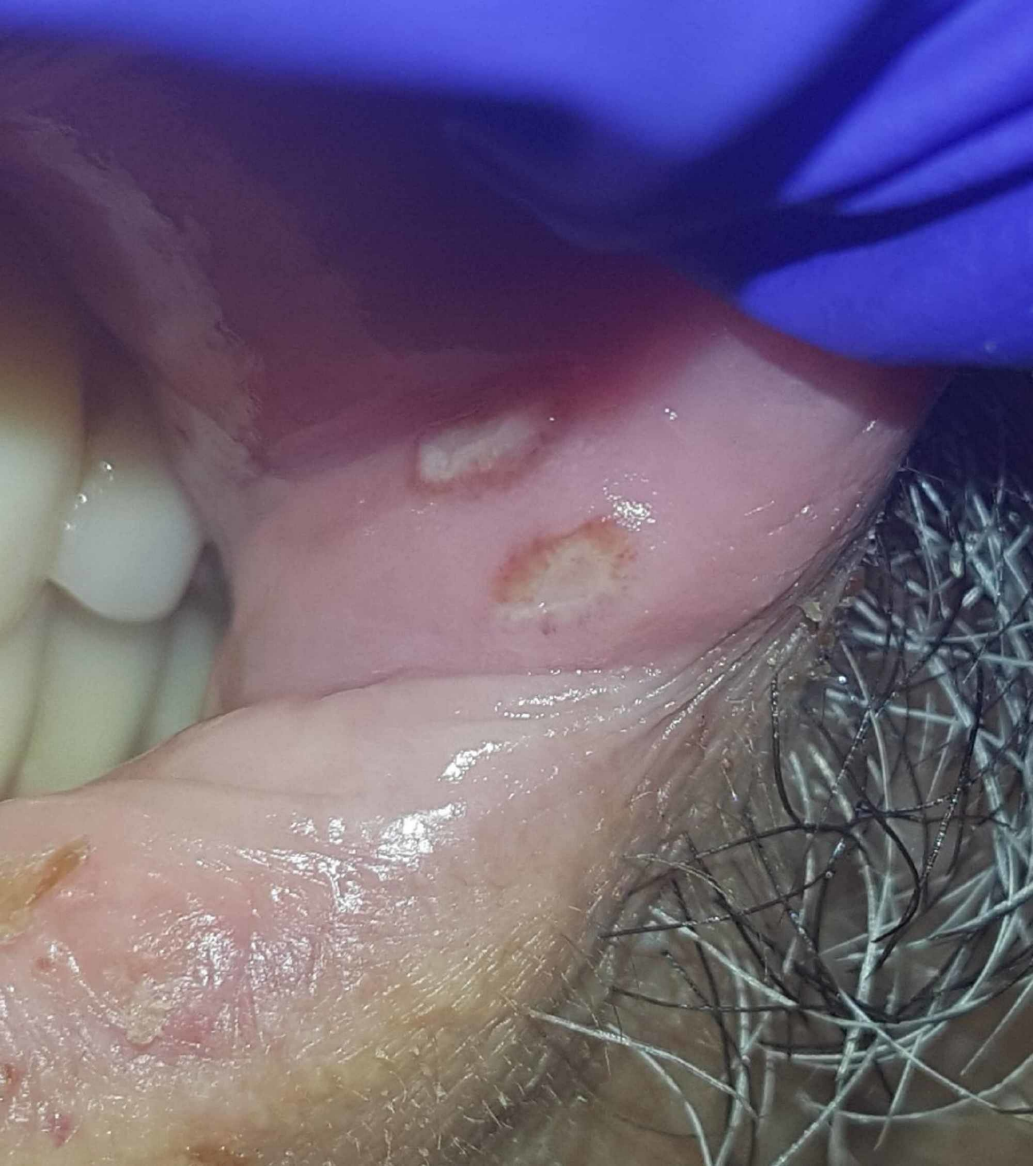
Oral ulcers with erythematous borders.

Based on this chest CT, he was initially thought to have an atypical pneumonia with possible septic emboli and he was started on intravenous antibiotics. His Cr increased from 1.74 mg/dL on hospital day (HD) 1 to 4.03 mg/dL on HD 5. His fractional excretion of sodium (FeNa) was 1%, fractional excretion of urea (FeUrea) was 38.3%, and 24-hour urine protein was 2.7 g, suggesting intrinsic renal disease. UA did not show casts or sediment. On HD 3, the patient developed debilitating polyarthritis with swelling and erythema involving his bilateral knees, shoulders, and eventually bilateral wrists (Figure 2). Synovial fluid analysis of the left knee joint was consistent with an inflammatory process (28,200 WBCs, 93% neutrophils, no crystals, negative culture). On HD 4, the patient developed oral ulcers with raised borders on his upper lip (Figure 3). On HD 5, he developed scant burgundy-colored hemoptysis, but respiratory status was otherwise stable.

Based on his history of chronic sinusitis, cavitary lung nodules with hemoptysis, new acute kidney injury with hematuria and proteinuria, migratory polyarthritis, and oral ulcers, he was clinically diagnosed with a small-vessel vasculitis, with suspicion for GPA, and started on high-dose intravenous methylprednisolone. However, due to his progressive renal failure, he then additionally received plasmapheresis and rituximab. Eventually, his labs revealed an ANCA titer of 1:320 with a cytoplasmic ANCA (c-ANCA) pattern and markedly elevated proteinase-3 (PR3) ANCA (420.6 IU/mL, Reference range <3.5 IU/mL). His antinuclear antibody, myeloperoxidase antibody, glomerular basement membrane antibody, rheumatoid factor, and anti-streptolysin O antibody were all within normal limits.

**Figure 4:**
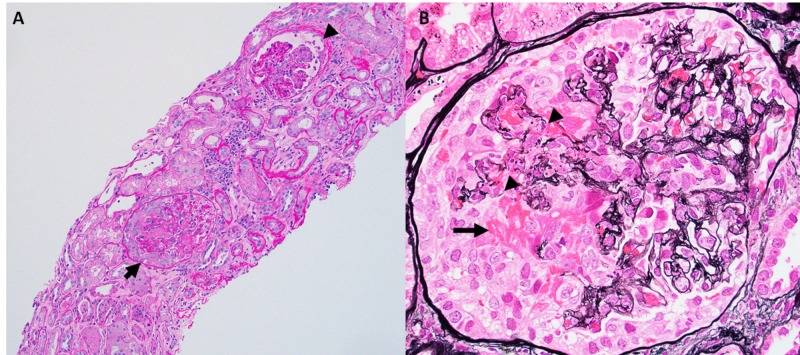
Necrotizing and crescentic glomerulonephritis (arrow). (A) Periodic acid Schiff stained biopsy section demonstrating glomeruli with a necrotizing lesion (arrow head) and a necrotizing cellular crescent. There is also increased interstitial inflammation and patchy acute tubular injury. (B) High magnification view of Jones methionine silver stained biopsy section demonstrating a necrotizing cellular crescent with disruption of underlying glomerular basement membranes (arrow heads) and extravasation of cells and fibrin (arrow) into the urinary space.

A renal biopsy (Figure 4) was performed on HD 9, which demonstrated 19 glomeruli (five globally sclerotic), with diffuse involvement (75%) of a severely active necrotizing and crescentic glomerulonephritis, as well as associated tubulointerstitial inflammation, mild parenchymal scarring (~10%), and severe arteriosclerosis. The immunofluorescence demonstrated a pauci-immune pattern with weak smudgy staining of the reactants in areas of capillary loop necrosis. Electron microscopic studies demonstrated glomerular basement membrane disruption and fibrin accumulation with only rare small electron-dense deposits. A diagnosis of severely active pauci-immune, PR3-ANCA-associated glomerulonephritis was made.

Despite this treatment, his renal function persistently declined, and he required hemodialysis, which he continued to require after hospitalization. The patient ultimately passed away several months after discharge from septic shock in the setting of Staphylococcus aureus bacteremia.

## Discussion

GPA is an AAV which classically presents with upper airway and pulmonary lesions, as well as renal injury. Our patient’s recurrent sinusitis and waxing and waning pulmonary symptoms with radiographic findings were highly suspicious for GPA. The most common symptoms at the time of presentation of GPA are ear, nose, and throat involvement (70-100%), pulmonary involvement (50-90%), and renal involvement (40-100%) [5]. The American College of Rheumatology (ACR) diagnostic criteria for the clinical diagnosis of GPA include having two or more of the following: nasal/oral inflammation, abnormal chest radiograph, abnormal urinary sediment, and pathologic correlation [5]. It is possible that the patient’s disease may have been present at several earlier time-points, including during the time of his prior sinus surgeries for chronic sinusitis, during his lung cancer surveillance which showed waxing and waning nodules, and at his first ED visit where he was treated for community-acquired pneumonia. However, it is unclear if earlier treatment would have altered his clinical course. What this case does suggest is that a high-index of suspicion is necessary to avoid diagnostic delay for AAV’s.

A biopsy is highly recommended to confirm the diagnosis of GPA. Regardless of autoantibody seropositivity, treatment should not be delayed if there is a high index of suspicion for the disease, as about 10% of patients with GPA are ANCA negative [6]. Immunosuppressive treatments are the mainstay of therapy and can reduce long-term morbidity and mortality. Initial induction therapy can include steroids, cyclophosphamide, rituximab, and plasmapheresis [1-3]. Induction therapy with corticosteroids combined with rituximab or cyclophosphamide has led to a survival of >80% for patients with GPA [3].

## Conclusions

GPA can be a life-threatening AAV, with fatal complications such as renal and respiratory failure. Patients with GPA often have preceding history of nasopharyngeal and upper airway disease, and can present with fluctuating pulmonary infiltrates not consistent with infection or malignancy after workup. The prompt recognition and treatment of patients with GPA can prevent life-threatening complications and reduce mortality.
